# Emulsion Electrospinning as an Approach to Fabricate PLGA/Chitosan Nanofibers for Biomedical Applications

**DOI:** 10.1155/2014/475280

**Published:** 2014-02-13

**Authors:** Fatemeh Ajalloueian, Hossein Tavanai, Jöns Hilborn, Olivier Donzel-Gargand, Klaus Leifer, Abeni Wickham, Ayyoob Arpanaei

**Affiliations:** ^1^Department of Textile Engineering, Center of Excellence in Applied Nanotechnology, Isfahan University of Technology, Isfahan 84156-83111, Iran; ^2^Department of Materials Chemistry, Uppsala University, 75121 Uppsala, Sweden; ^3^Department of Engineering Sciences, Applied Materials Science, Uppsala University, 75121 Uppsala, Sweden; ^4^Department of Physics, Chemistry and Biology, Division of Molecular Physics, Linkoping University, 58183 Linkoping, Sweden; ^5^Department of Industrial and Environmental Biotechnology, National Institute of Genetic Engineering and Biotechnology, Tehran 14965-161, Iran

## Abstract

Novel nanofibers from blends of polylactic-co-glycolic acid (PLGA) and chitosan have been produced through an emulsion electrospinning process. The spinning solution employed polyvinyl alcohol (PVA) as the emulsifier. PVA was extracted from the electrospun nanofibers, resulting in a final scaffold consisting of a blend of PLGA and chitosan. The fraction of chitosan in the final electrospun mat was adjusted from 0 to 33%. Analyses by scanning and transmission electron microscopy show uniform nanofibers with homogenous distribution of PLGA and chitosan in their cross section. Infrared spectroscopy verifies that electrospun mats contain both PLGA and chitosan. Moreover, contact angle measurements show that the electrospun PLGA/chitosan mats are more hydrophilic than electrospun mats of pure PLGA. Tensile strengths of 4.94 MPa and 4.21 MPa for PLGA/chitosan in dry and wet conditions, respectively, illustrate that the polyblend mats of PLGA/chitosan are strong enough for many biomedical applications. Cell culture studies suggest that PLGA/chitosan nanofibers promote fibroblast attachment and proliferation compared to PLGA membranes. It can be assumed that the nanofibrous composite scaffold of PLGA/chitosan could be potentially used for skin tissue reconstruction.

## 1. Introduction

Tissue engineering as a means of functional tissue fabrication or repair requires three-dimensional (3D) scaffolds that provide the structural base (matrix) for cell attachment and proliferation [1]. Among different methods to produce 3D scaffolds, electrospinning has received considerable interest [2, 3]. This technique allows fabrication of porous nanofibrous webs with specific characteristics like three dimensional morphology, large surface area to volume ratio, and high porosity. In addition, the structure of electrospun nanofibrous webs mimics the size range of natural extracellular matrix (ECM) fibrous components. ECM provides a natural base for cell adhesion, proliferation, migration, and metabolism [4–6].

Electrospinning has been used to fabricate nanofibrous scaffolds from synthetic and natural polymers. The most frequently electrospun synthetic polymers include glycolide- and lactide-based linear aliphatic polyesters [7, 8]. Although such synthetic polymers are biodegradable and have good mechanical properties, they suffer from poor cell-scaffold attachment interactions due to their hydrophobic structure as a result of lower surface energy [1]. Additionally, their negative surface charge and acidity cause product degradation [9]. These shortcomings have restricted the application of linear aliphatic polyesters in pure form for scaffold purposes.

Collagen, elastin, chitosan, and alginate in the pure form are examples of naturally derived polymers that have been electrospun for the production of nanofibrous webs [2, 10–14]. Among these polymers, chitosan, a naturally occurring polysaccharide, and its derivatives have been widely explored for biomedical applications, thanks to their biocompatibility and biodegradability [15, 16]. A number of studies have been performed to evaluate the cytocompatibility of chitosan using a variety of cell types such as osteoblasts, fibroblasts, hepatocytes, and neural cells. These studies have shown that chitosan is nontoxic and can support growth of the aforementioned cell types [17, 18]. Like many natural polymers, chitosan suffers from poor mechanical properties [19]. In addition, it is difficult to electrospin chitosan in its pure form. This is mainly due to the limited number of solvents for chitosan as well as high viscosities at its low concentrations [20–22].

Recently, mixture of polymers has attracted a great deal of attention for producing polyblend nanofibers, exhibiting a combination of characteristics related to each polymer employed in the so-called polyblend [2]. Basically, a polyblend nanofibrous scaffold containing synthetic and natural polymers is expected to exhibit physicochemical properties of both components like hydrophilicity/hydrophobicity, surface charge, and mechanical strength of synthetic polymers as well as the biochemical signatures of natural fibers [1, 23]. Considering different properties of PLGA and chitosan, a nanofibrous scaffold of the polyblend of PLGA/chitosan should show interesting applications in tissue engineering.

Literature reports several efforts aiming to produce electrospun scaffolds containing both PLGA and chitosan. For example, some researchers have used two sets of separate syringe pumps and power supplies with a common collector to produce a web consisting of a mixture of individual PLGA and chitosan nanofibers [24, 25] or a coaxial electrospinning setup to produce a mat of core-sheath type nanofibers to electrospin with PLGA as the core and chitosan as the sheath [26]. Other researchers have used a common solvent for both the polymers [27]. This approach restricts the fraction of one polymer in the solution due to limitations of simultaneous solubility of the two polymers. A recent report also introduces electrospinning of PLGA/chitosan nanofibers in which PLGA is grafted by chitosan [28].

The present study aimed at developing an approach for preparation of nanofibrous scaffolds of PLGA/chitosan through an emulsion electrospinning technique. In this way, there is no need to use specially designed electrospinning setups as already explained. Moreover, there is no need for a common solvent for both polymers or grafting one of the polymers onto the other one. Such a scaffold is expected to be mechanically strong, because of PLGA, as well as hydrophilic as a result of the presence of chitosan. The hydrophilicity bestows the mat a surface prone to cell attachment. The novelty of this approach is applying the method of emulsion electrospinning [29] to produce polyblend nanofibers from synthetic and natural polymers with varying ratios. The challenge of trying to have an electrospinnable mixture of water insoluble PLGA and water soluble chitosan made us choose an emulsion system. The selected emulsifier was polyvinyl alcohol (PVA). Not only is PVA a rather easy electrospinnable polymer, but it also dissolves in water and hence it can, if required, be extracted from the polyblend nanofibers.

## 2. Experiment

### 2.1. Materials

Chitosan (MW *≈* 200,000 g/mol, degree of deacetylation *≈* 90%) and PLGA (MW 40,000–70,000 g/mol, L-lactide/glycolide, 50 : 50) were purchased from Aldrich (Germany). PVA (MW *≈* 72,000 g/mol), chloroform (analytical grade), dimethylformamide (DMF) (analytical grade), glacial acetic acid, and hexamethyldisilazane (HMDS) were obtained from Merck (Germany). DMEM (Dulbecco's Modified Eagle Medium 1x), trypsin/EDTA, and phosphate-buffered saline (PBS) were purchased from GIBCO Invitrogen, USA. Fetal bovine serum (FBS Hyclone research grade) was bought from Perbio Scientific, Sweden. CellTiter 96 AQueous One solution was purchased from Promega, Madison, WI, USA. 3T3 fibroblast cell line was purchased from American Type Culture Collection (ATCC). All solvents were used as received without further purification.

### 2.2. Preparation of the Electrospinning Emulsion

PLGA solutions (12%, 16%, 20% w/v) were prepared by dissolving PLGA in chloroform/DMF (volume ratio of 90 : 10) at 50°C under magnetic stirring for 3 h. Chitosan solutions (4%, 6% w/v) were prepared by dissolving chitosan in acetic acid (14%) at room temperature. Also, a PVA solution (8% w/v) in double distilled water was prepared at 90°C.

To obtain optimum conditions for emulsion electrospinning, a series of emulsions containing different ratios of PLGA and chitosan were prepared (Table 1). The emulsions were prepared by simultaneous adding of PVA and chitosan solutions to PLGA solution, followed by mixing with magnetic stirrer. The volume ratio of the 3 solutions was 1 : 1 : 1 in all the emulsions. The final mixtures were stirred at room temperature for 12 h to obtain a homogenous emulsion.

### 2.3. Electrospinning

Electrospinning of the emulsions was carried out, with a voltage of 14–16 kV applied to the blunt needle (21 gauge) tip of the 1 mL syringe (filled with the emulsion) and the grounded aluminum foil collector. The feed rate was 0.25 mL/h. The needle tip to collector distance was 15 cm. The same electrospinning conditions were used to electrospin pure PLGA (16% w/v in chloroform/DMF (volume ratio of 90 : 10)) as a reference for electrospun mats of PLGA/chitosan.

### 2.4. Surface Morphology of Electrospun Nanofibers

The surface morphology of electrospun PLGA/chitosan nanofibers was investigated with the help of scanning electron microscope (SEM) (Seron Technology AIS 2100) micrographs. The average fiber diameter of the electrospun fibers was measured by applying Image J software to the SEM micrographs. Statistical analysis was done using SPSS 8.0. (SPSS Inc. Chicago, USA). Transmission electron microscopy (TEM) (JEOL 2000 FXII (2401)) was employed to investigate the miscibility of PLGA and chitosan. To obtain TEM micrographs of the cross section of nanofibers, electrospun samples were embedded directly in EPON 812 (epoxy resin) first and then polymerized at 60°C. Sections were cut with the diamond knife of microtome, floated on water, and collected on Cu-grids. The sections were contrasted with 2% uranyl acetate and Reynolds lead solution afterwards. TEM pictures were converted with Gatan software Digital Micrograph 3.6.5.

### 2.5. PVA Extraction

To extract the PVA from the electrospun nanofibers with the aim of obtaining nanofibers containing only PLGA and chitosan, five samples of PLGA/chitosan/PVA (sample Number 2 in Table 1) with a thickness of about 100 *μ*m were cut (20 mm × 20 mm), weighed (±0.1 mg), and then immersed in an aqueous solution containing ethanol (50% v/v) at room temperature for 8 h. The samples were finally weighed after drying overnight in a vacuum oven under room temperature.

### 2.6. Water Contact-Angle Measurement

The degree of hydrophilicity of PLGA, PLGA/chitosan/PVA, and PLGA/chitosan electrospun samples was determined with the help of a video-based optical contact angle meter (DataPhysics OCA 15EC). Small pieces (4 × 1 cm) of the samples were cut and placed on a glass microscope slide using double-sided tape to ensure uniform viewing of the surface. The glass slide was placed on the stage and a 6 *μ*L drop was placed on the sample surface. The contact angle on the left and right sides of the drop was measured by SCA software. The average of ten angles is reported for each sample.

### 2.7. Mechanical Properties

The tensile properties of the electrospun samples with a thickness of 50–100 *μ*m (60 mm × 10 mm) were determined by the ZWICK Z005 testing machine (ZWICK, Germany) equipped with a 50 N load-cell. The cross-head speed and the gauge length were 10 mm/min and 40 mm, respectively. The average of five measurements is reported. To compare the strength of samples in wet condition with that of dry ones, wet specimens were prepared by soaking the samples in phosphate buffered saline (PBS) for 8 h at 37°C.

### 2.8. *In Vitro* Degradation

The degradability tests were performed in phosphate buffered saline solution (PBS) adjusted to pH 7.4 at 37°C. Electrospun PLGA and PLGA/chitosan mats were cut into 1 cm × 1 cm pieces, vacuum-dried at room temperature, and weighed accurately. They were then placed into closed vials containing 10 mL of PBS and were kept in water bath (37°C) under mild shaking. At each time point (1, 4, 7, 14, 21, 28, 42, and 56 days), three samples of each type of PLGA or PLGA/chitosan were removed from PBS solution, rinsed with distilled water, and completely dried under vacuum at room temperature. The degradation rate was reported as the loss in the mass of electrospun mat using the formula *“*mass  loss  (%) = ((*M*_0_ − *M*_*t*_)/*M*_0_) × 100,” where *M*_0_ and *M*_*t*_ are the mass of electrospun mat before and after incubating in PBS solution, respectively.

### 2.9. FT-IR Analysis

FT-IR spectrophotometer (Perkin Elmer Spectrum 100) was used to record the FT-IR spectra of pure chitosan, electrospun mats of PLGA, PLGA/chitosan/PVA, and PLGA/chitosan nanofibers. The range of 650–4000 cm^−1^ with a resolution of 4.0 cm^−1^ and 32 scans in transmission mode was employed.

### 2.10. Differential Scanning Calorimetry (DSC) Analysis

To evaluate the state of PLGA/chitosan miscibility in the emulsion-electrospun nanofibers, DSC (TA instruments, Q1000, TA Instruments Ltd., USA) was employed. DSC analysis was carried out for pure chitosan powder, pure PVA powder, and electrospun mats of PLGA, PLGA/chitosan/PVA and PLGA/chitosan. A cool-heat-cool cycle was applied at a rate of 5°C/min, starting from 30°C and increasing to 200°C, and then cooling to −50°C. The given DSC thermograms were obtained during reheating the samples to 200°C under nitrogen atmosphere.

### 2.11. Cell Culture of 3T3 Fibroblasts

NIH 3T3 mouse fibroblasts were cultured in DMEM (Dulbecco's Modified Eagle Medium, Gibco, USA) containing 10% fetal bovine serum (FBS Hyclone research grade, Perbio Scientific, Sweden), 50 U/mL penicillin, and 50 U/mL streptomycin (Sigma-Aldrich, Sweden). The medium was replaced every 3 days and cultures were maintained in a tissue culture incubator at 37°C with 5% CO_2_. After reaching about 80% confluence, cells were detached by 0.05% trypsin/0.05% EDTA. Electrospun mats (PLGA/chitosan and PLGA) were sterilized by ultraviolet irradiation for both top and bottom surfaces in a laminar flow hood (each side for 20 min); cells were seeded onto scaffolds under density of 5000 cells/cm^2^ and were placed in 24-well plates.

### 2.12. Metabolic Activity and Proliferation of 3T3 Fibroblasts

Cell viability and metabolic activity in response to different substrates of tissue culture polystyrene cover slip (TCP) and electrospun samples of PLGA and PLGA/chitosan were measured using MTS cytotoxicity assay (CellTiter 96 AQueous Non-Radioactive Cell Proliferation Assay, Promega, Madison, WI, USA), according to the manufacturer's instructions. Cells were seeded at a density of 5000 cells/cm^2^ and the cell activity was evaluated during a 7-day period. On days 1, 4, and 7, the cell seeded electrospun samples were washed with PBS and transferred to new well plates containing 500 *μ*L of culture medium in each well. Then, 100 *μ*L of MTS solution was added to each well. Cells were maintained for an additional 4 hours in a humidified incubator at 37°C and 5%   CO_2_. The absorbance was read at 450 nm in an ELISA plate reader (Multiskan Plus, LabSystem, Finland) and the response was defined as (cell-seeded scaffolds A450 − mean blank)/(mean TCP 1 day A450 − mean blank) ∗ 100%, where mean blank is culture medium absorbance mean. In this way, cell metabolic activity on each substrate in different time points was compared with TCP on day 1. This was done in triplicates followed by calculations of mean values and standard deviations. Cell proliferation was determined by counting the number of DAPI-stained cell nuclei that were associated with the PLGA or PLGA/chitosan nanofibers. Cell nuclei were counterstained with 4,6-diamidino-2-phenylindole dihydrochloride (DAPI, Sigma-Aldrich; 1 *μ*g/mL) in three separate samples (PLGA or PLGA/chitosan) per time points of 1, 4, and 7 days. A minimum of 5 randomly selected visual fields (at 20 × magnification in a fluorescent confocal microscope, Zeiss LSM 510, Germany) were studied.

### 2.13. Cell Morphology

Morphological study of 3T3 fibroblasts grown on electrospun PLGA and PLGA/chitosan nanofibers was performed after 1 and 7 days of cell culture by SEM. Cell-seeded nanofiber constructs were harvested and washed by PBS and subsequently fixed in 2.5% glutaraldehyde for 3 h. The scaffolds were dehydrated with increasing concentrations of ethanol (30%, 50%, 70% 90%, and 100%) for 10 min each. Finally the cell-scaffold constructs were treated with hexamethyldisilazane (HMDS) to further water extraction. The dehydrated, cell-seeded constructs were maintained in desiccators equipped with a vacuum for overnight air drying. After sputter-coating with Platinum, SEM was used to observe cell and scaffold morphology and cell attachment on nanofiber scaffolds.

### 2.14. Statistical Analysis

All the data presented are expressed as mean ± standard deviation (SD) of the mean. Statistical analysis was performed using a one-way analysis of variance (ANOVA) using Student's *t*-test to determine the statistical significance between the two means evaluated at *P* < 0.05.

## 3. Results and Discussion

To study the possibility of having nanofibrous scaffolds of PLGA/chitosan with different ratios of chitosan to PLGA in final mats from a single electrospinning setup, 6 emulsions were tested (Table 1). Having a water soluble polymer (chitosan) and a water insoluble polymer (PLGA), an emulsion mixture could be the key to do successful electrospinning. PVA was used as emulsifier and our preliminary tests revealed that the best concentration of PVA is 8% (w/v) which was kept constant for all emulsions.

### 3.1. Electrospinning

**Figure 1:**
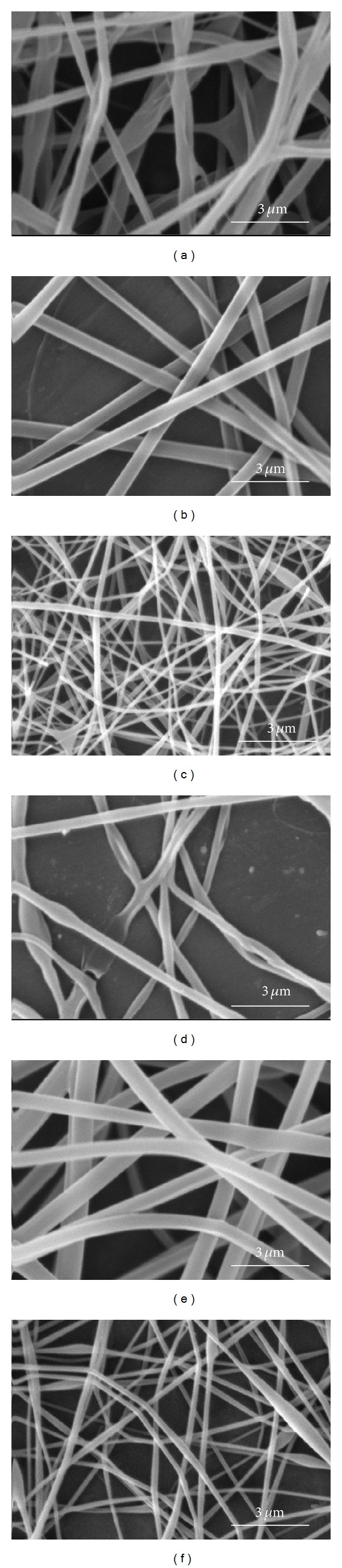
SEM images of emulsion electrospun PLGA/Chitosan/PVA nanofibrous mats ((a), (b), (c), (d), (e), and (f) are SEM images of samples numbered as 1, 2, 3, 4, 5, and 6 described in Table 1, respectively. Electrospinning parameters are voltage of 16 Kv, feed rate of 0.25 mL/h, and tip-collector distance of 15 cm).

**Figure 2:**
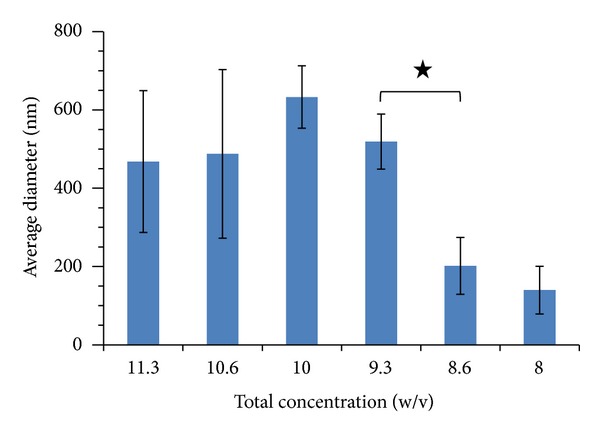
Variation of the average diameter of the electrospun nanofibers (prior to PVA extraction) versus the total emulsion concentration.

**Figure 3:**
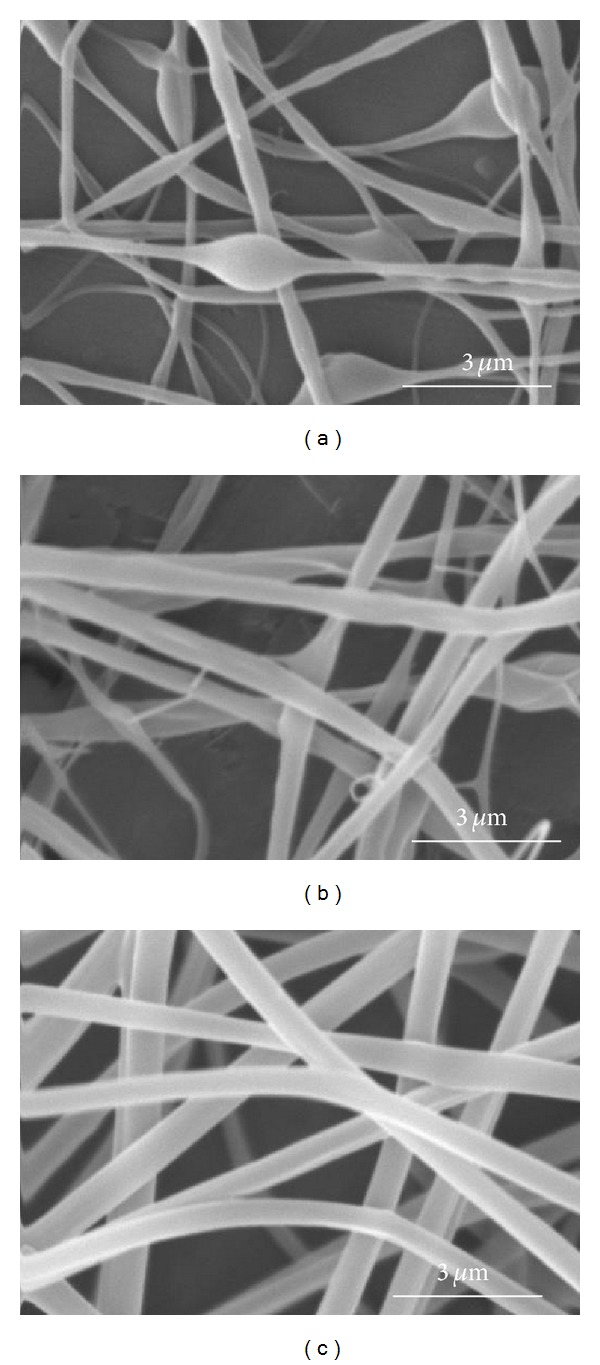
SEM images of PLGA (16%)/chitosan (4%)/PVA (8%) nanofibrous mats prepared using various voltages of (a) 8 kV, (b) 12 kV, and (c) 16 kV (feed rate = 0.25 mL/h, tip to collector distance = 15 cm).

To find optimum electrospinning conditions, several factors of emulsion concentration, applied voltage, feed rate, and tip to collector distance were tried. The SEM images in Figure 1 show the morphology of nanofibers electrospun from the 6 emulsions shown in Table 1 with the electrospinning conditions mentioned in the caption. The variation of the average diameter of the electrospun nanofibers (prior to PVA extraction) versus the total concentration of each emulsion (containing PLGA, chitosan, and PVA) is shown in Figure 2. As can be seen, the average diameter of electrospun nanofibers decreases with lower total concentration of polymers in the emulsion. This can be related to the lower viscosity of emulsions as a result of lower polymer concentration. Images in Figure 1 and the plot in Figure 2 also lead to the conclusion that decreasing the concentration of PLGA in the emulsion from 16% to 12% decreases the average diameter sharply. This is related to the higher share of chitosan in the emulsion. Similar results have been reported by Meng et al. who electrospun PLGA/gelatin and observed a sharp decrease in the diameter of nanofibers as a result of lowering the ratio of PLGA relative to gelatin [30]. Our work showed that concentration of PLGA in the range of 12% to 16% and the concentration of chitosan in the range of 4% to 6% in the electrospinning emulsions led to optimum electrospinning. Higher concentrations of PLGA and chitosan produced emulsions which were too viscous for electrospinning. SEM images of PLGA (16%)/chitosan (4%)/PVA (8%) nanofibers electrospun at voltages of 8, 12, and 16 kV are shown in Figure 3. Other electrospinning conditions are mentioned in the corresponding caption. Evidently, at 8 kV (Figure 3(a)), beads are formed along the electrospun nanofibers. Increasing the voltage to 12 kV leads to the disappearance of beads, but the nanofibers are less uniform (Figure 3(b)). However, voltage of 16 kV produces uniform nanofibers (Figure 3(c)). It can be concluded that a stronger electrical field resulting from higher voltage induces a higher draw in the emulsion jet trajectory leading to the formation of bead-free, more uniform, and finer PLGA/chitosan/PVA nanofibers. As far as needle tip to collector distance during electrospinning of PLGA (16%)/chitosan (4%)/PVA (8%) is concerned, the results show that, with voltage of 16 kV and feed rate of 0.25 mL/h, increasing the needle tip to collector distance from 15 cm to 25 cm decreases the average nanofiber diameter from 519 ± 70 nm to 502 ± 84 nm which is practically insignificant. Literature also shows that needle tip to collector distance in the range of 8–15 cm has no significant effect on the average diameter of electrospun PVA [31] and chitosan [32] nanofibers. Electrospinning of the emulsion of PLGA (16%)/chitosan (4%)/PVA (8%) nanofibrous mats with feed rates of 0.15 mL/h and 0.25 mL/h (needle tip to collector distance = 15 cm, voltage = 16 kV) led to the fabrication of nanofibers with average diameters of 369 ± 120 nm and 519 ± 70 nm, respectively. As expected, lowering the feed rate led to a lower average diameter, but a lower uniformity of the electrospun nanofibers was observed.

### 3.2. PVA Extraction

**Figure 4:**
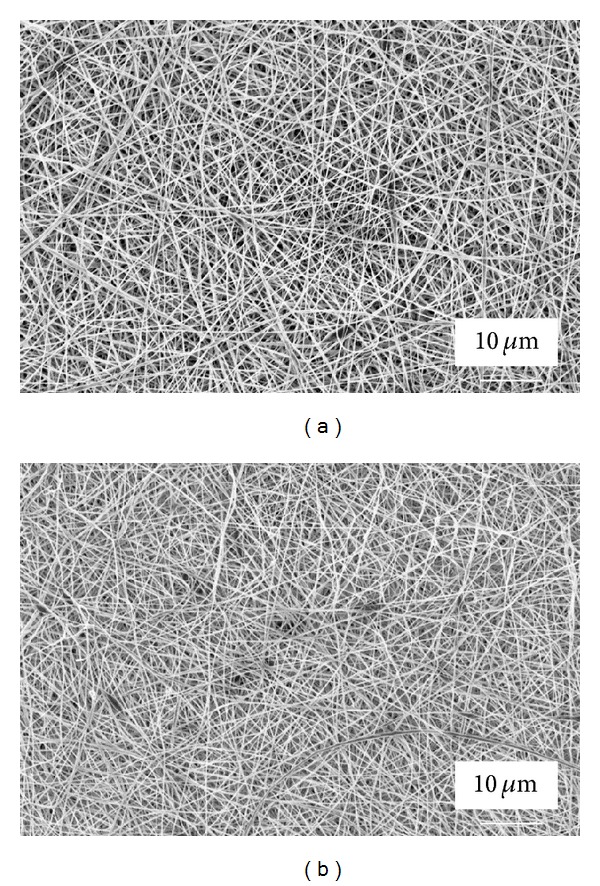
PLGA (16%)/chitosan (4%)/PVA (8%) electrospun mats: (a) before and (b) after PVA extraction in ethanol 50%-water 50% for 8 hours.

Considering the purpose of this work, that is, the fabrication of a nanofibrous mat of PLGA/chitosan, PVA was removed from electrospun mats. This increases the share of the natural polymer (chitosan) in the final mat as well as leading to a more porous structure [33, 34]. As Table 1 shows, the share of chitosan in the final electrospun nanofibers (after PVA extraction) varies from 16% to 33%. To remove PVA from the electrospun polyblend nanofibrous mats, an aqueous solution containing 50% ethanol was used. Mass reduction measurements showed that 8 h was sufficient for the PVA removal. We found that 100% water is not a suitable solvent, as chitosan has a water solubility of about 10% [35]. However, PVA dissolves slightly in ethanol and chitosan solubility in water decreases almost linearly with increasing the share of ethanol in a water-ethanol mixture, so that the solubility of chitosan in the solution of water-ethanol (50-50) is less than 0.1% [35].  Figure 4 shows the electrospun sample of PLGA (16%)/chitosan (4%)/PVA (8%) before and after PVA extraction. This sample (sample number 2 in Table 1) has been used for further analyses of FT-IR, contact angle measurement, tensile testing, *in vitro* degradation, DSC, TEM, and cell seeding and is referred hereafter to as PLGA/chitosan/PVA prior to PVA removal and PLGA/chitosan after PVA removal.

### 3.3. Hydrophilicity

**Figure 5:**
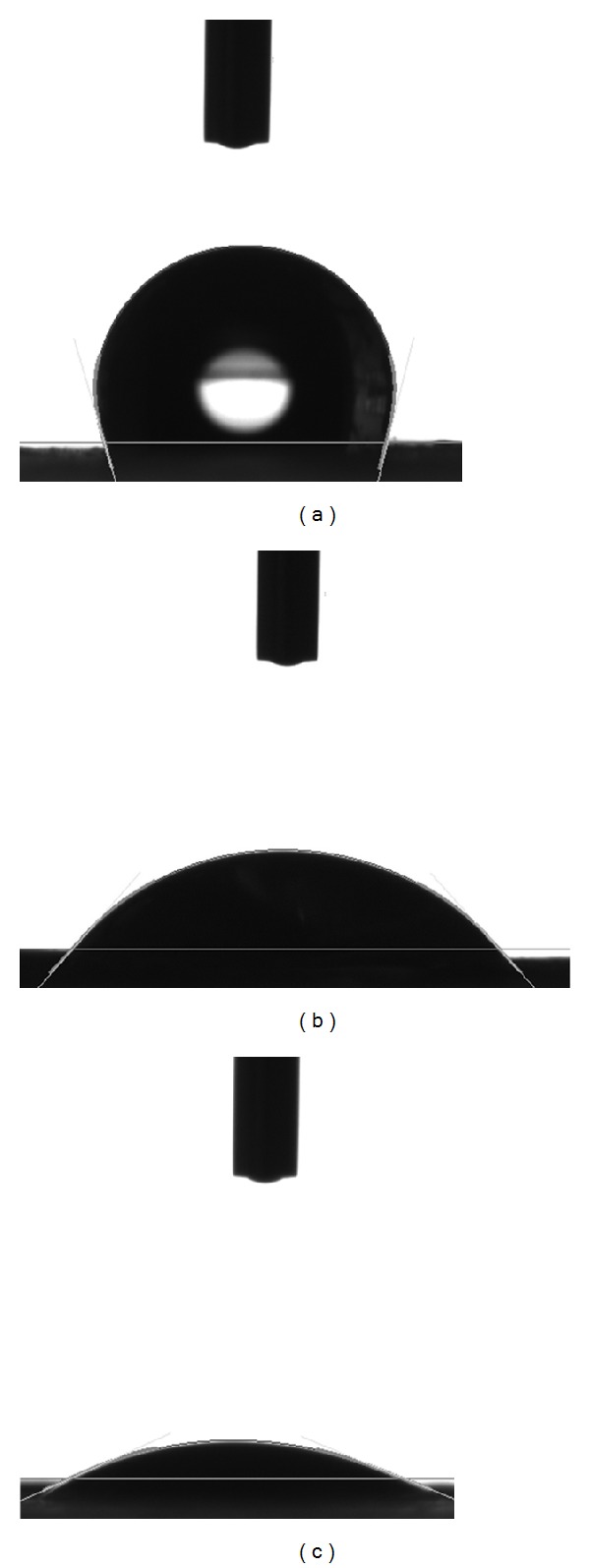
Contact angle of (a) PLGA, (b) PLGA/chitosan/PVA, and (c) PLGA/chitosan electrospun mats.

Water contact angles of pure PLGA, PLGA/chitosan/PVA, and PLGA/chitosan mats are shown in Figure 5. The average contact angle of PLGA (105.5 ± 5.2°), PLGA/chitosan/PVA (48 ± 7.1°), and PLGA/chitosan (24 ± 8.7°) reveals the hydrophobic nature of PLGA mat (Figure 5(a)) as well as the role of chitosan in bestowing hydrophilicity to the PLGA/chitosan nanofibrous mat. Also, it can be seen that the removal of PVA from the electrospun nanofibers leads to higher hydrophilicity (Figures 5(b) and 5(c)), which has been attributed to the generation of more pores in the composite membrane after PVA dissolution [34]. The improvement in hydrophilicity of the PLGA/chitosan scaffold is expected to lead to higher cell affinity of the hybrid scaffold compared to PLGA [36, 37].

### 3.4. Tensile Properties

**Figure 6:**
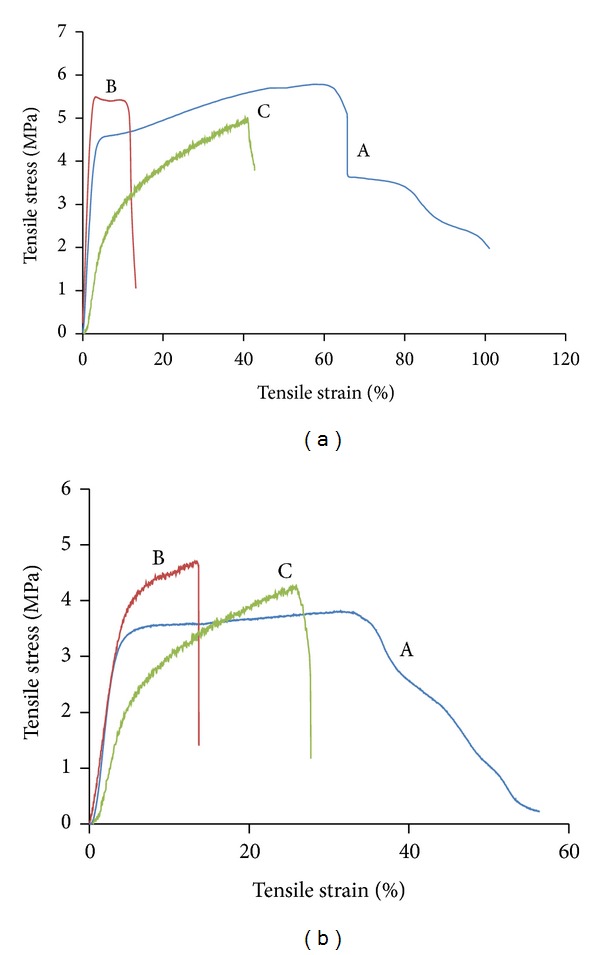
Tensile strength curves of (A) PLGA, (B) PLGA/chitosan/PVA, and (C) PLGA/chitosan electrospun mats under (a) dry and (b) wet conditions.

Figure 6 compares the tensile characteristics of PLGA, PLGA/chitosan/PVA, and PLGA/chitosan in dry (Figure 6(a)) and wet (Figure 6(b)) conditions. For dry condition, addition of chitosan to PLGA leads to a strong reduction in elongation at break, whereas there are no significant differences in Young's modulus or tensile strength. It is also observed that PVA removal has no effect on tensile strength but results in a sharp decrease in Young's modulus from 274.3 ± 17.2 MPa in PLGA/chiotsan/PVA to 64 ± 11.8 MPa in PLGA/chitosan. However, the PVA removal has led to an increase in extensibility of the hybrid structure, leading to more flexibility of the PLGA/chitosan, compared to PLGA/chitosan/PVA. It could be due to the PVA extraction which causes a longitudinal shrinkage in PLGA/chitosan construct (around 30%). In the wet condition, there is an overall decline in tensile characteristics of all three samples as expected, whilst pure PLGA exhibits a higher reduction.

### 3.5. *In Vitro *Degradation

**Figure 7:**
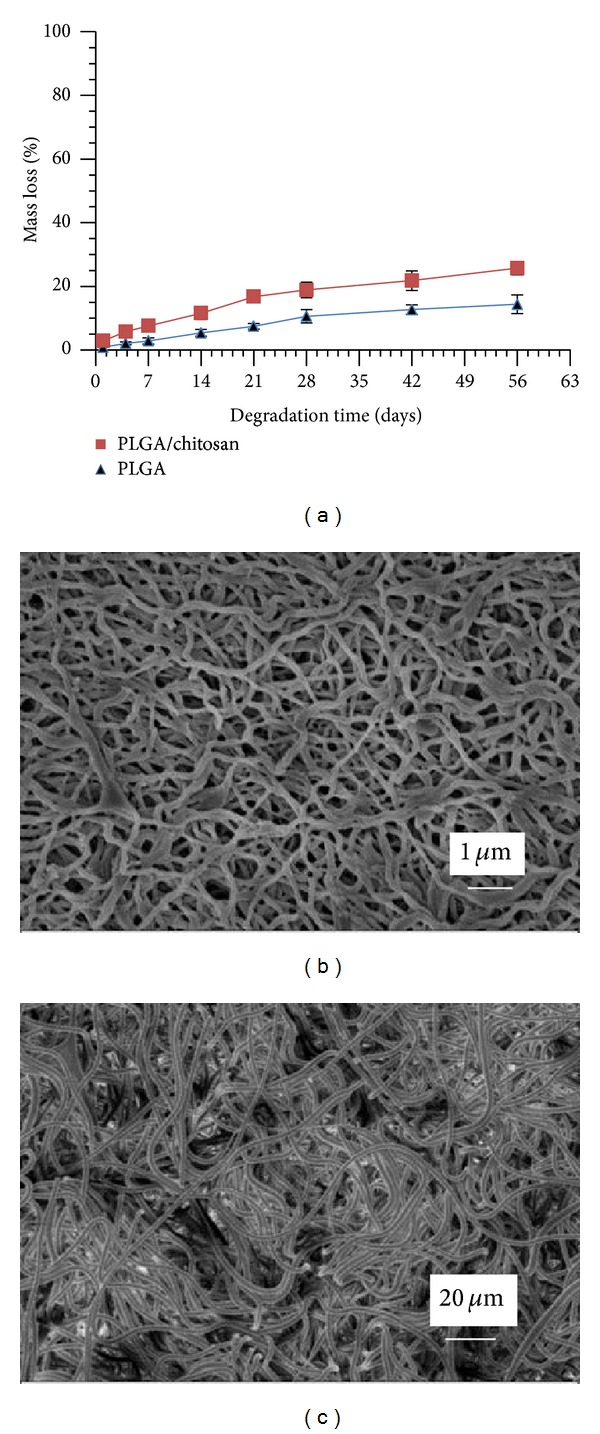
Degradation of PLGA/chitosan and PLGA nanofibers: (a) comparing the mass loss percentage at different time points, (b) and (c) SEM image of PLGA/chitosan and PLGA nanofibers, respectively, after 14 days of incubation in PBS solution at 37°C.

To simulate the *in vivo* degradation, we have studied the hydrolysis *in vitro* under physiological conditions at pH 7.4 using PBS. We observed a higher mass loss for the polyblend nanofibrous mat (PLGA/chitosan) in all time points in comparison with PLGA (Figure 7(a),). The morphological changes of both the mat types, after two weeks of incubation in PBS during *in vitro* degradation, are shown in Figure 7(b) (PLGA/chitosan) and Figure 7(c) (PLGA). It is seen that the fibrous morphology in the PLGA mat was almost unchanged, whilst the fibers in the composite mat were somehow swollen. However, no obvious morphological changes in both scaffolds were observed up to 8 weeks. These results suggest that both the PLGA and PLGA/chitosan nanofibers can be considered as potential scaffolds for biomedical applications like skin tissue engineering where the rate of tissue formation can be around several weeks [38, 39].

### 3.6. FT-IR Spectra

**Figure 8:**
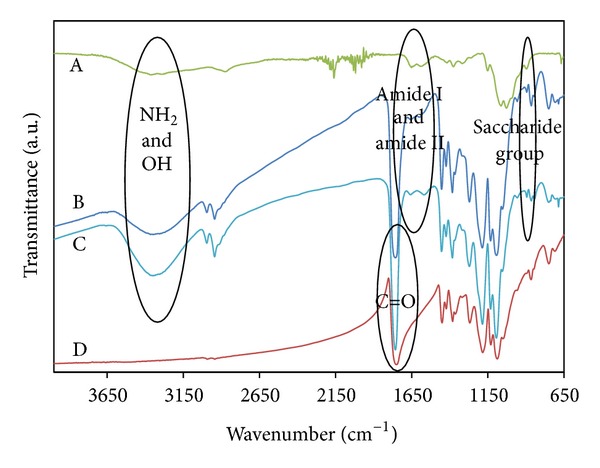
FTIR spectra of (A) pure chitosan and electrospun mats of (B) PLGA/chitosan, (C) PLGA/chitosan/PVA, and (D) PLGA.

The FT-IR spectra of pure chitosan, pure PLGA, PLGA/chitosan/PVA and, PLGA/chitosan are shown in Figure 8. The characteristic absorption bands of PLGA lie at about 2922 cm^−1^ (asymmetric CH_2_ stretching), 2853 cm^−1^ (symmetric CH_2_ stretching), 1752 cm^−1^ (C=O stretching), 1452 cm^−1^ (C–H stretching), 1182 cm^−1^ (C–O–C stretching), and 1130 cm^−1^ (C–O stretching) [38]. Conversely, the characteristic absorption bands of chitosan lie at 1154 cm^−1^ and 893 cm^−1^ (saccharide groups), 1535 cm^−1^ (N–H bending), 1600 cm^−1^ (amide I stretching), 1647 cm^−1^ (amide II bending), 2977 cm^−1^ (C–H stretching), and 3425 cm^−1^ (N–H stretching) with the broad peak between 3400 and 3700 cm^−1^, corresponding to the stretching of O–H [40, 41]. Comparing the above mentioned spectra with those of the PLGA/chitosan/PVA and PLGA/chitosan nanofibers leads to the conclusion that both PLGA and chitosan are present in the polyblend emulsion electrospun nanofibers. Moreover, extraction of PVA leads to a narrower and shorter peak in the range of 3400–3700 cm^−1^ which originates from fewer hydroxyl groups after PVA removal.

### 3.7. Issue of Miscibility or Immiscibility

**Figure 9:**
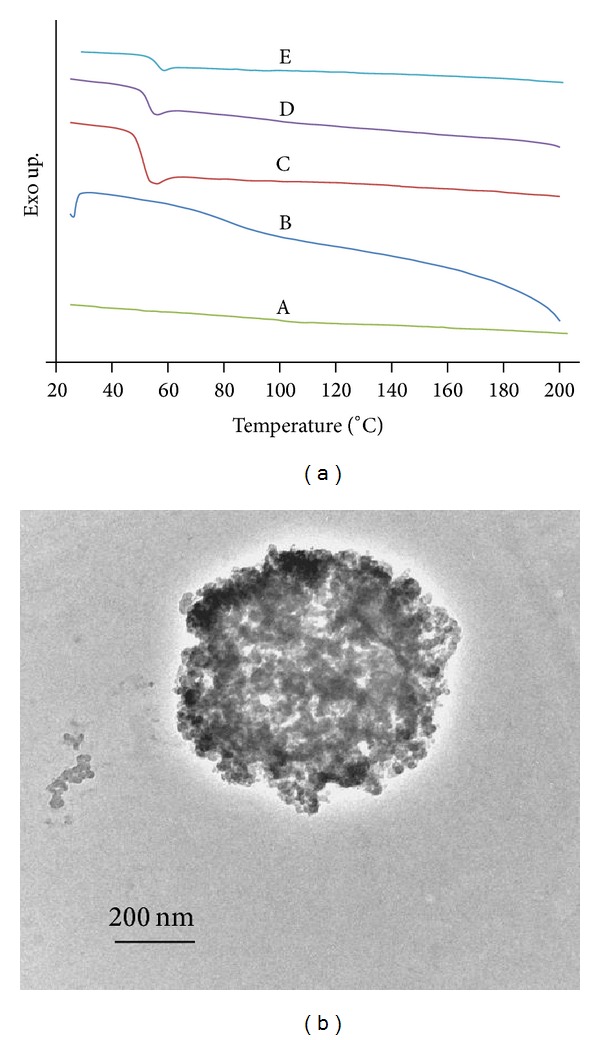
Miscibility evaluation of PLGA and chitosan in electrospun polyblend of PLGA/chitosan based on (a) DSC thermograms of (A) pure chitosan, (B) pure PVA, and electrospun mats of (C) PLGA, (D) PLGA/chitosan/PVA, and (E) PLGA/chitosan; and (b) TEM image of the cross section of PLGA/chitosan/PVA electrospun sample embedded in EPON.

DSC and TEM were employed to assess the phase miscibility of PLGA/chitosan nanofibers produced by electrospinning. DSC thermograms of the second heat run for pure chitosan, PVA powder, electrospun mats of PLGA, PLGA/chitosan/PVA and., PLGA/chitosan are shown in Figure 9(a). As can be seen, the glass transition temperature (*T*_*g*_) of chitosan is around 153°C which is in accordance with that reported by other researchers [42–44]. The additional inclination of DSC curve appearing at about 92°C for chitosan can be explained by the water-induced relaxation [44]. The *T*_*g*_ of PLGA and PVA are 45.2°C and 74.8°C, respectively. As can be seen, the DSC curves of PLGA/chitosan/PVA and PLGA/chitosan mats show a *T*_*g*_ of 47°C and 48.2°C, respectively. Moreover, no additional peaks are observed when compared with the thermograms of pure PLGA, PVA, and chitosan. The very small shift of *T*_*g*_ in PLGA/chitosan/PVA and PLGA/Chitosan blends relative to the *T*_*g*_ of pure materials indicates nonmiscible blends as declared by Wang et al. [45]. However the absence of any considerable inclination around *T*_*g*_ of PVA, in the DSC thermogram of PLGA/chitosan/PVA, suggests miscibility of PVA and chitosan [46]. These DSC results imply that PLGA and chitosan have poor miscibility. In spite of the conclusions reached from DSC thermograms, TEM cross-sectional image of PLGA (16%)/chitosan(4%)/PVA(8%) fibers (Figure 9(b)) indicates a heterogeneous structure with homogenous distribution of PLGA and chitosan which suggests some miscibility between PLGA and chitosan. It is worth mentioning that, although a clear contrast originating from the segregation of the two polymers cannot be observed in Figure 9(b), the image indicates that the system is multiphase. Considering the results of DSC and TEM, the system is better described as microphase separated. Hence, the term described by Utracki as “immiscible but compatible” blends can be applied to the situation. Such a blend conforms to definitions given for compatibility (a visibly homogeneous mixture) and immiscibility (a blend that does not conform to the thermodynamic conditions of phase stability) [45].

### 3.8. Cell Metabolic Activity and Proliferation

**Figure 10:**
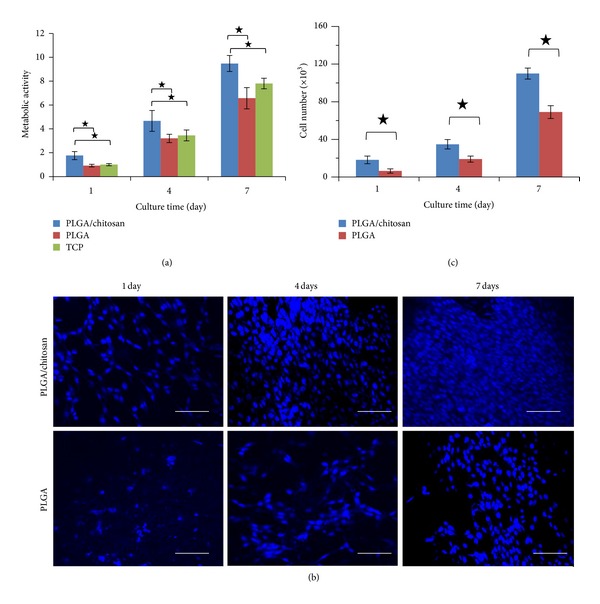
Viability and proliferation of PLGA/chitosan and PLGA nanofibers: (a) metabolic activity of 3T3 fibroblasts on PLGA/chitosan and PLGA nanofibers and TCP measured by an MTS assay, (b) DAPI-stained nuclei of 3T3 fibroblasts on PLGA/chitosan, and PLGA nanofibers after 1, 4, and 7 days; scale bar representing 100 *μ*m, and (c) proliferation of 3T3 fibroblasts on PLGA/chitosan and PLGA nanofibers measured by counting number of cell nuclei stained by DAPI.

We used murine cell lines (NIN 3T3 fibroblast) to evaluate the biocompatibility of our scaffolds. Considering the fact that 99% of mouse genes have an equivalent in humans, mice are widely used as a model [47] for function of human cells/genes in different studies [48, 49]. The metabolic activity of 3T3 fibroblasts on the scaffolds prepared by electrospinning was determined by an MTS assay after culturing the cells on the nanofibers over a period of 7 days (Figure 10(a)). It was found that the metabolic activity of cells on PLGA and PLGA/chitosan nanofibers increases with culture time, similar to the trend observed on tissue culture plates (TCP). PLGA/chitosan nanofibers demonstrated higher number of viable cells in all time points compared to PLGA nanofibers and TCP. A significant increase was also found from first day of culture onto both PLGA/chitosan and PLGA nanofibers to four and seven days after culturing. Cell proliferation studies were performed by counting the number of adhered cells (cell nuclei stained by DAPI) on PLGA and PLGA/chitosan nanofibers. It was found that there is a progressive trend in number of cells adhered to both PLGA and PLGA/chitosan scaffolds after 1, 4, and 7 days of culturing (Figures 10(b) and 10(c)). As the rate of proliferation on PLGA/chitosan scaffold was significantly higher in all time points, compared to PLGA, we conclude that PLGA/chitosan represents better cell interactions and would be a good candidate for skin regeneration applications. As mentioned earlier, the PLGA/chitosan composite scaffold is expected to provide the necessary mechanical support to the tissue to be replaced (here skin) and offer cellular cues to assist in the regeneration of the target tissue. Incorporating the hydrophilic polymer of chitosan into PLGA has successfully triggered the biological responses such as cellular proliferation and metabolic activity in PLGA/chitosan compared to PLGA scaffold, which is consistent with previous reports [18, 50]. Rather than higher cell adhesion and viability observed with the polyblend nanofiber of PLGA/chitosan, antibacterial properties of chitosan [51, 52] as well as its potential to initiate fibroblast proliferation [53] (due to gradual release of N-acetyl-*β*-D-glucosamine during the degradation procedure) make it an appropriate candidate for applications like wound healing and skin regeneration. It should also be considered that chitosan is a hemostat and can lead to natural blood clotting, which restricts its application in several fields of regenerative medicine. However, it is a positive point for wound healing, since the formation of a clot serves as a temporary shield which protects the denuded wound tissues and provides a provisional matrix over and through which cells can migrate during the repair process [54].

### 3.9. Cell Morphology

**Figure 11:**
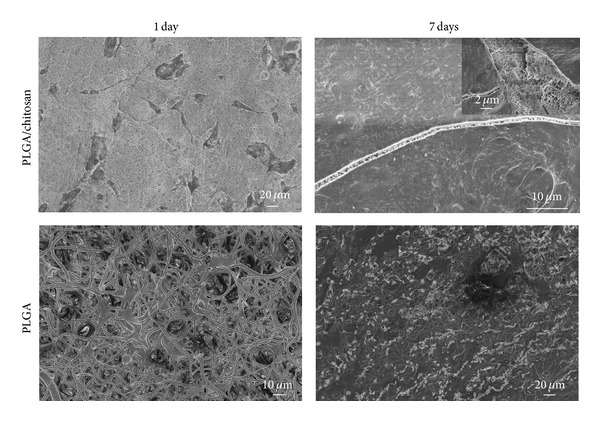
SEM images of 3T3 fibroblasts after 1 and 7 days culturing onto PLGA/chitosan and PLGA nanofibers.

The SEM micrographs of fibroblasts cultured on PLGA and PLGA/chitosan nanofibers for 1 and 7 days are represented in Figure 11. It can be seen that fibroblasts attached on both the membranes (after 1 day) and changed their original round shape to elongated shape. However, they have covered the surface of scaffolds after 7 days, making a multilayer in PLGA/chitosan scaffold (the image on the corner of PLGA/chitosan 7 days), confirming the higher proliferation rate of fibroblasts on PLGA/chitosan nanofibers.

## 4. Conclusion

A method for producing electrospun PLGA/chitosan mat from a single setup has been presented which alleviates the need for two sets of separate electrospinning systems or a coaxial electrospinning setup. Moreover, the possibility of having different PLGA to chitosan ratios in the PLGA/chitosan nanofibers has been confirmed by SEM images of electrospun mats obtained from homogenous emulsions of PLGA/chitosan/PVA. The polyblend structure is much more hydrophilic than pure PLGA which is a key point for improved cell-scaffold interactions. FT-IR spectra show no chemical interaction between PLGA and chitosan. Mechanical tests show that the nanofibrous mats of PLGA/chitosan enjoy enough strength in both dry and wet conditions for many biomedical applications. DSC, SEM, and TEM results indicate that the emulsion system has been capable of introducing a compatible, homogenously distributed mixture of PLGA and chitosan in PLGA/chitosan nanofibers. We observed that both the electrospun PLGA and PLGA/chitosan scaffolds promote the fibroblast attachment and proliferation. Favorable interaction between cell-cell and cell-matrix was also demonstrated by cell morphology seen in SEM images. Considering higher rate of viability and proliferation in PLGA/chitosan nanofibers, it can be assumed that such a composite scaffold fabricated through emulsion electrospinning would be an appropriate candidate for skin tissue engineering applications.

## Figures and Tables

**Table 1:** The content of emulsion electrospinning solutions and the electrospun nanofibers.

Sample number	PLGA conc. (w/v)	Chitosan conc. (w/v)	PVA conc. (w/v)	Total emulsion conc. (w/v)	PLGA (%W) after and before PVA removal	Chitosan (%W) after and before PVA removal
1	20	4	8	10.6	84 (62.5)	16 (12.5)
2	16	4	8	9.3	80 (57)	20 (14.3)
3	12	4	8	8	75 (50)	25 (16.7)
4	20	6	8	11.3	77 (60)	23 (17.6)
5	16	6	8	10	73 (53)	27 (20)
6	12	6	8	8.6	67 (46)	33 (25)
